# Requirements for nucleocapsid-mediated regulation of reverse transcription during the late steps of HIV-1 assembly

**DOI:** 10.1038/srep27536

**Published:** 2016-06-07

**Authors:** Pierre-Jean Racine, Célia Chamontin, Hugues de Rocquigny, Serena Bernacchi, Jean-Christophe Paillart, Marylène Mougel

**Affiliations:** 1 Centre d'études d’agents pathogènes et biotechnologies pour la santé, CPBS-CNRS, Université de Montpellier, 1919 Route de Mende, 34293 Montpellier, France; 2 Laboratoire de Biophotonique et Pharmacologie, UMR 7213 CNRS, Faculté de Pharmacie, Université de Strasbourg, 74 Route du Rhin, 67401, Illkirch Cedex, France; 3 Architecture et Réactivité de l’ARN, Université de Strasbourg, CNRS, IBMC, 15 rue René Descartes, 67084, Strasbourg, France

## Abstract

HIV-1 is a retrovirus replicating within cells by reverse transcribing its genomic RNA (gRNA) into DNA. Within cells, virus assembly requires the structural Gag proteins with few accessory proteins, notably the viral infectivity factor (Vif) and two copies of gRNA as well as cellular factors to converge to the plasma membrane. In this process, the nucleocapsid (NC) domain of Gag binds to the packaging signal of gRNA which consists of a series of stem-loops (SL1-SL3) ensuring gRNA selection and packaging into virions. Interestingly, mutating NC activates a late-occurring reverse transcription (RT) step in producer cells, leading to the release of DNA-containing HIV-1 particles. In order to decipher the molecular mechanism regulating this late RT, we explored the role of several key partners of NC, such as Vif, gRNA and the cellular cytidine deaminase APOBEC3G that restricts HIV-1 infection by targeting the RT. By studying combinations of deletions of these putative players, we revealed that NC, SL1-SL3 and in lesser extent Vif, but not APOBEC3G, interplay regulates the late RT.

Retroviruses such as HIV-1 possess the property to convert their genomic RNA (gRNA) into DNA after entry into target cells. This reverse transcription (RT) is proceeding during ongoing disassembly of the viral particle and trafficking to the nucleus where the DNA copy will integrate into the host chromosomes. The RT takes place within a large RNP complex whose composition and location in the cell is a source of debate. It contains the gRNA, viral reverse transcriptase, integrase (IN), matrix (MA), capsid (CA), nucleocapsid (NC), viral Protein R (VpR) and viral infectivity factor (Vif) (for review^1^,^2^).

The role of NC in RT has been well documented with an emphasis on its nucleic acid-binding and chaperoning activities mainly held by two highly conserved zinc finger (ZF) motifs (for a review^3^). *In vitro* studies showed that NC chaperones primer tRNA annealing to the primer binding site (PBS) and the primer activation signal (PAS) of gRNA, and the two obligatory strand transfers that are required to generate the complete proviral DNA (for review^4^). In cells, mutating the first (ZF1) or the second (ZF2) motif leads to a decrease of viral DNA stability and a drastic reduction of subsequent DNA integration^5^. Interestingly, the two ZFs are not equivalent. Indeed, the NC mutant in which ZF1 has been duplicated (ZF1+ZF1) is more replication competent than mutants with zinc finger-swap (ZF2+ZF1) or with duplicated ZF2 (ZF2+ZF2)^6^. Several studies of ZF mutants revealed that ZF1 was more critical than ZF2 for the NC chaperone activity^7^,^8^,^9^. In addition, nucleic acid binding abilities of different NC domains have been analyzed and revealed that the absence of ZF2 (NC 1-35) did not reduce NC binding affinity, demonstrating that ZF1 provides the major contribution to DNA binding^9^.

Like NC, Vif is a small basic protein (23 kDa) that harbors RNA chaperone activity implicated in the initiation of RT^10^,^11^,^12^. Vif behaves as a cofactor by stimulating the binding of reverse transcriptase and primer^13^, increasing the polymerization rate of the reverse transcriptase^14^, facilitating the bypass of abasic sites^14^ and modulating the post-penetration stability of the RNP complex^15^,^16^. The major role of Vif relies in the neutralization of a potent intracellular innate defense factor that protects mammals from viral DNA invasion in their genome^17^. By counteracting the antiviral activity of cellular cytidine deaminases, mainly APOBEC3G (A3G) and A3F, Vif prevents the newly made DNA from an intense deamination during RT that is associated with a strong reduction of viral infectivity^18^,^19^,^20^,^21^,^22^.

Interestingly, NC also modulates RT during the late steps of HIV replication. Indeed, RT should be restricted to avoid that DNA synthesis starts before virus release producing DNA-containing particles. At this stage, NC acts as a repressor of RT since mutating the NC promotes an extensive RT in producer cells. Then, these cells released particles containing DNA reverse-transcripts^23^,^24^,^25^. A recent study of the biogenesis of DNA-containing HIV-1 by live-cell total internal-reflection fluorescent (TIRF) microscopy showed that production of DNA-containing viruses was a consequence, and not the cause, of budding defects^26^. Furthermore, genetic and biochemical studies of the DNA-virus biogenesis revealed a deficiency in Tsg101 recruitment, a key component of the cellular ESCRT-I machinery. These data indicated that NC and Tsg101 interplay restricts the DNA synthesis in the producer cells, and favors gRNA packaging into viruses, thus supporting a functional link between RT and late assembly^26^. The occurrence of RT during virus assembly is a natural property of the foamy viruses which was named “late RT” in contrast to the “early RT” initiated after foamy virus entry^27^. Based on the similarities with the foamy viruses, we use the term of late RT to refer to HIV-1 RT taking place during the late steps of assembly.

Despite the requirement of an intact NC in *in vitro* RT, it is puzzling how the late RT could be efficient in the case of NC ∆ZF2-deleted HIV-1. Actually, the complex molecular mechanism regulating the late RT remains largely unknown. A possible link with Gag and GagPol processing, which occurs concomitantly with virus assembly and budding is not straightforward. Indeed, blocking the processing of Gag and GagPol in NC mutants (with the combination of protease and NC mutations) decreased the late RT activity without elimination since late RT activity remained 500-fold higher than WT^23^. Analysis of Pol processing in ∆ZF2-producing cells and virions did not reveal significant modification^26^. Finally, no direct correlation between late RT and polyprotein processing was found with the well-characterized HIV-1 CA WM184*/*185AA mutant. These substitutions in the CA dimer interface affected infectivity, diminished immature particle production, and impaired Gag assembly and precursor protein processing (accumulation of cleavage intermediates)^28^,^29^,^30^. Nevertheless, the defects in WM184/185AA Gag processing were not sufficient to confer and activate late RT activity in producer cells^26^. Therefore, defects in assembly and maturation did not necessarily lead to the activation of late RT. Then, the link between late RT and maturation appears to be more complicated than expected.

Here, to gain a better understanding on the molecular mechanism of the late RT, we first determine the putative role of key partners of NC.

Efficiency and specificity of gRNA packaging rely on recognition of the gRNA by the viral Gag proteins. Gag through its NC domain binds to the packaging signal (Psi) located next to the PBS in the highly structured and multifunctional 5′ untranslated region (UTR) of the gRNA^31^,^32^,^33^,^34^. Psi folds into three stem-loops (SL1 to SL3) with SL1 and SL3 acting as the main packaging determinants (for review^35^,^36^). Since Psi constitutes the primary binding site of NC, it was interesting to determine whether the NC activity in late RT regulation relies on RNA-NC interaction and whether late RT could be activated by disrupting NC-Psi interaction without mutating NC. As an RNA-binding protein, Vif also interacts with the 5′-end region of gRNA and contributes to HIV-1 RNA dimerization^12^,^37^,^38^,^39^,^40^,^41^. Vif seems also to plays an active role in virus assembly and behaves as an integral component of viral particles^42^.

The two NC and Vif proteins are both acting in the early and late steps of the HIV-1 replication cycle. The two RNA-chaperone proteins play key roles in RT, RNA binding and virus assembly (for review^21^). During assembly, Gag and Vif colocalize in producer cells and interact with each other *via* the Gag NC domain and the C-terminus of Vif 41,43. Therefore, we hypothesized that Vif could be a cofactor of NC and both proteins could act *via* gRNA interactions as temporal regulator of the late RT. The roles of Vif and Psi have never been investigated in the context of late RT. Thus, combined with *in vitro* analysis of the chaperone activity of Vif and NC variants in RT, we measured the activity of Vif in RT by biochemical analysis of the producer cells and released virions. By establishing a stable cell line expressing the antiviral A3G factor, we provided clear evidence that Vif could modulate the late RT independently on its function in A3G neutralization. Importantly, we showed that deletions in Psi sequence resulted in activation of late RT even in the presence of WT NC, indicating that NC requires the integrity of its primary RNA-binding site to restrict the late RT.

## Results

### NC-mediated late RT in producer cell and rationale of the study

We and the Gorelicks team previously demonstrated that mutating HIV-1 NC lead to the release of DNA-containing particles^23^,^24^,^25^. Viral DNA synthesis was already completed before virus release from cells because the intravirion DNA level was insensitive to virus incubation with or without dNTP or treatment with the Nevirapine, a HIV-1 RT inhibitor. In agreement with this conclusion, Nevirapine pre-treatment of the producer cells prevented the presence of DNA in released particles^23^,^25^. More directly, newly made reverse-transcripts were found in the producer cells under the forms of strong-stop cDNA, full-length DNA or cDNA copies of viral spliced RNAs, and importantly their levels were dependent on Nevirapine treatment, demonstrating that DNA synthesis did occur in producer cells and not inside viral particles. Note that during these experiments, perfect co-variations in DNA levels were observed between the spliced and other DNA forms^25^. Although spliced cDNA species are much less abundant than the other forms of viral DNAs, they constitute useful markers of reverse transcriptase activity. Indeed, viral spliced RNAs are reverse transcribed as efficiently as the gRNA^44^ and the presence of spliced cDNA resulted unambiguously from RT^25^. In contrast, it is difficult to discriminate by qPCR between transfected DNA plasmid (pNL4-3) and viral cDNA molecules originating from gRNA template.

**Figure 1:**
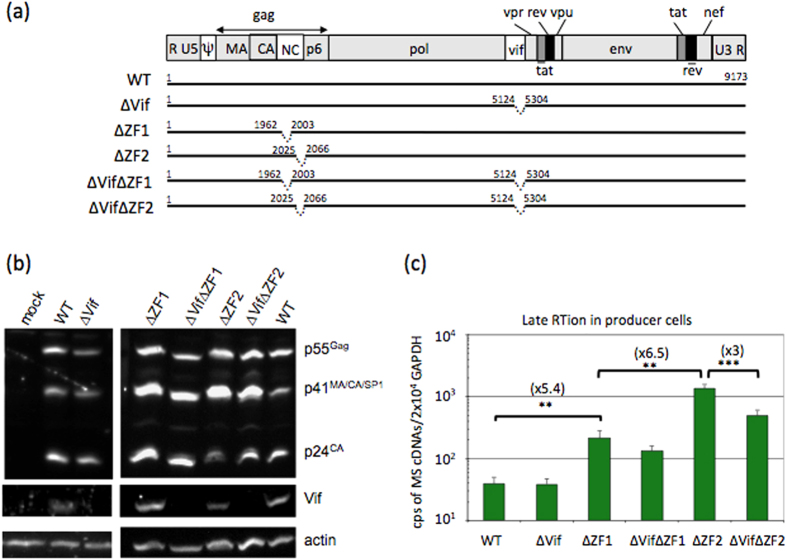
Effect of NC and/or Vif deletions on late RT activity in producer cells. (**a**) Schematic representation of mutants used in this study. The organization of the HIV-1 genome was depicted with deletions and numbers according to NL4-3. (**b**) Representative western blot analysis of three independent experiments showing Gag, its processed products and Vif in HEK 293T cells transfected or not with wild-type (WT) and mutant HIV-1 constructs. Proteins were detected as described in Materials and Methods. The input cell lysates were monitored with anti-actin antibody. (**c**) Quantitation of the late RT activity in producer cells. Amount of reverse transcripts generated from viral MS mRNAs was determined by qPCR. The number of MS cDNA copies (cps) was measured in at least three independent transfection experiments and normalized to the cellular GAPDH gene. ^*^^*^^*^P < 0.001, ^*^^*^P < 0.01.

Considering late RT, similar phenotypes were observed between subtle substitutions or complete deletions of the ZFs of NC. In both cases, RT activations were stronger when mutating the distal ZF (ZF2) rather than mutating the proximal one (ZF1) (Fig. 1a). Since the presence of intravirion DNA directly depends on late RT activities in producer cells, their levels are not depending on the levels of co-packaged RNAs that do not serve as templates in virions.

First, we determined the levels of spliced cDNAs in cells transfected with HIV-1 missing either ZF1 or ZF2 motif of NC. These two NC mutants are known to release DNA-containing particles^25^, but the intracellular cDNA rate has not been determined in the ∆ZF1-producing cells. The two NC mutants were efficiently expressed in cells as monitored by Gag western blot (WB) analysis (Fig. 1b). As previously reported^26^,^45^,^46^, mutating the NC ZF motifs modified Gag processing as evidenced by the accumulation of p41^MA/CA/SP1^ precursor (Fig. 1b). The levels of multi-spliced (MS) cDNAs were determined by specific qPCR. As expected, increased cDNA levels were measured with the two NC mutants compared to WT. In agreement with the previous experiments performed with subtle substitutions in each ZFs, the highest DNA level was achieved with the ∆ZF2 mutant indicating that the two ZFs in a wild-type context prevent the late RT activity in the producer cells (Fig. 1c).

### Vif activates the late RT when the ZF2 of NC is absent

**Figure 2:**
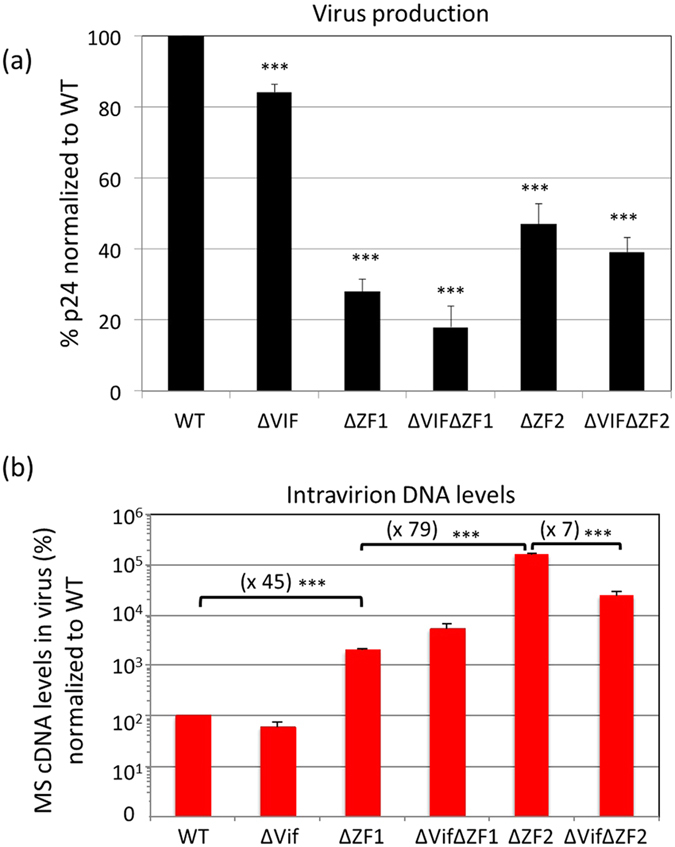
Effect of NC and/or Vif deletions on virus production and content. (**a**) Quantitative analysis of virus production. Culture fluids of producer cells (same cells as in Fig. 1) from at least three independent transfections were concentrated and the amount of viruses was determined by p24 ELISA. Values were expressed as percentage of WT HIV-1 level. (**b**) DNA content of viruses. Intravirion levels of MS DNAs were determined by qPCR normalized to WT. ^*^^*^^*^P < 0.001.

To determine whether the viral Vif protein participates to the late RT process in the producer cells, we compared the effects of Vif-deleted virus in the context of restricted (WT) or activated (∆ZFs) late RT in cells. Vif was deleted from the WT (pNL4-3∆Vif) and from the two NC mutants (pNL4-3∆Vif ∆ZF1 and pNL4-3∆Vif ∆ZF2) (Fig. 1a). Since Vif has been shown to modulate the proteolytic processing of Gag precursor^47^,^48^, we controlled that the Vif deletions did not modify the Gag patterns of the respective parental constructs as monitored by WB (Fig. 1b). Vif deletion did not significantly modify the levels of MS cDNAs in cells producing HIV-1 WT or ∆ZF1 (Fig. 1c). In contrast, DNA synthesis activity of ∆ZF2 was 3-fold reduced when Vif was deleted, revealing a contribution of Vif in the late RT when the second ZF motif is absent. This contribution of Vif should have consequences in the DNA content of released virions. First, virus production was monitored by a p24 ELISA on pelleted viral particles. Virus level was reduced with all tested mutations, ranging from 20 to 80% reduction of virus production (Fig. 2a). Then, copy numbers of intravirion MS cDNAs were determined by qPCR and normalized to a same amount of viral particles (Fig. 2b). The intravirion MS cDNA levels showed no modification in the presence or the absence of Vif (for WT and ∆ZF1 viruses), except in the ∆ZF2 context where virions contained 7-time less cDNA when Vif is absent (compared to ∆ZF2). Similar effects were observed when the DNA levels were normalized to the levels of RNA genome inside virions (Supplemental Fig. S1a). This decrease resulted from the reduced cDNA amount observed in ∆Vif∆ZF2 producer cells, supporting the fact that Vif and NC contributed to the control of late RT. As previously reported, but still unresolved, activation of late RT resulted in stronger effects in virion content than in producer cells^24^,^25^. These results suggest that Vif acts as an activator of the late RT when the ZF2 motif is missing.

### Effects of NC and Vif on *in vitro* RT

**Figure 3:**
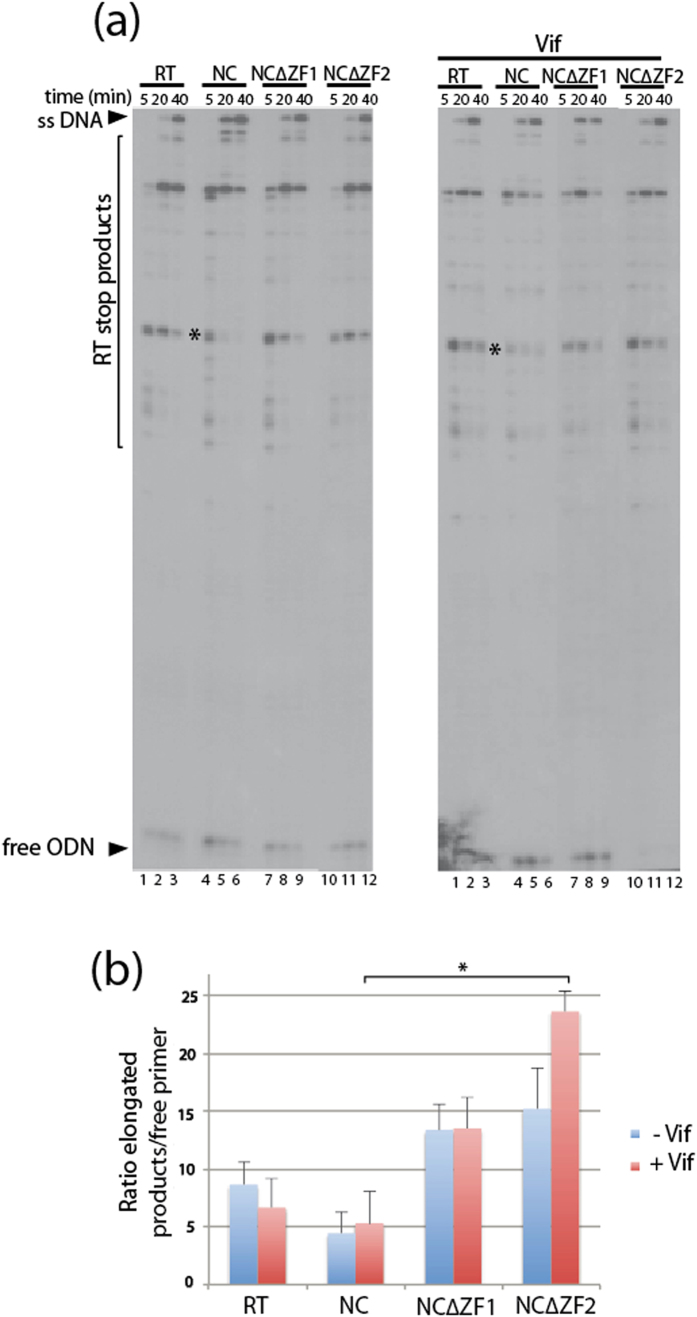
Effect of Vif and NC on *in vitro* reverse transcription. A DNA primer (ODN) heat-annealed to RNA 1-278 (NL4.3 isolate) was extended by incubating with HIV-1 RT and dNTPs in presence or absence of NC (saturating concentration, of 1.8 μM i.e. 1 NC/5 nts) and Vif (0.2 μM) proteins. Polymerization was for 5, 20, or 40 min and the samples were deproteinized before PAGE. (**a**) Two representative denaturing gels showing the time course of labeled ODN extension in the presence/absence of NC and/or Vif. Minus strand strong-stop (ss) DNA, RT stop products and unextended ODN (free ODN) are indicated. Asterisks indicate RT pauses specifically reduced by the addition of WT NC. (**b**) Gels were quantified using a FLA 5000 BioImager (Fuji) and histogram represents the ratio of elongated RT products (ssDNA+RT stop products)/unextended ODN primer at the final time point (40 min). Standard deviations are representative of at least three independent experiments. ^*^P < 0.05.

Mature NCp7 is a well characterized co-factor of HIV-1 RT, and at least some of the Vif binding sites in the 5′ region of viral RNA are also NC binding sites^49^. While the role of NC on cDNA synthesis has been well studied, surprisingly the effects of deleting either one of the two NC ZFs in RT have been poorly documented and the combined effects of Vif and NC on this step have been only partially analyzed^12^. In a goal to correlate the observed cellular effects with NC and Vif constructs (Figs 1 and 2), we performed *in vitro* extension experiments at a NC protein concentration corresponding to complete coverage of the RNA template (1 NC/5 nts) in order to mimic late RT conditions in which the NC is maintained at high concentrations with respect to viral gRNA and Vif at low concentration (Fig. 3). When we compared, without Vif, the cDNA synthesis in the presence of NC WT or ZF mutants (Fig. 3a, left panel), we first observed that all proteins were able to induce the synthesis of cDNA. However, the remaining free ODN primer (unextended) slightly decreased for both NC mutants compared to WT NC as indicated by the increased ratio of elongated products/free primer (Fig. 3b, blue bars), suggesting an improved initiation of RT when the ZFs of NC are missing. As previously observed^12^, WT NC protein was able to reduce RT stops induced by strong secondary structures of RNA (Fig. 3, asterisk). Interestingly, while the presence of Vif did not influence the general behavior of RT with WT NC (Fig. 3a right panel), it did increase the total fraction of elongated cDNA products for the two NC ZF mutants and very few free ODN primer was remaining, especially for the ∆ZF2 mutant (Fig. 3b, red bars). Taken together, these results suggest that Vif positively contributed to increase the RT process when the ZFs of NC are deleted.

### A3G does not influence the effect of Vif and NC on DNA accumulation into virion.

**Figure 4:**
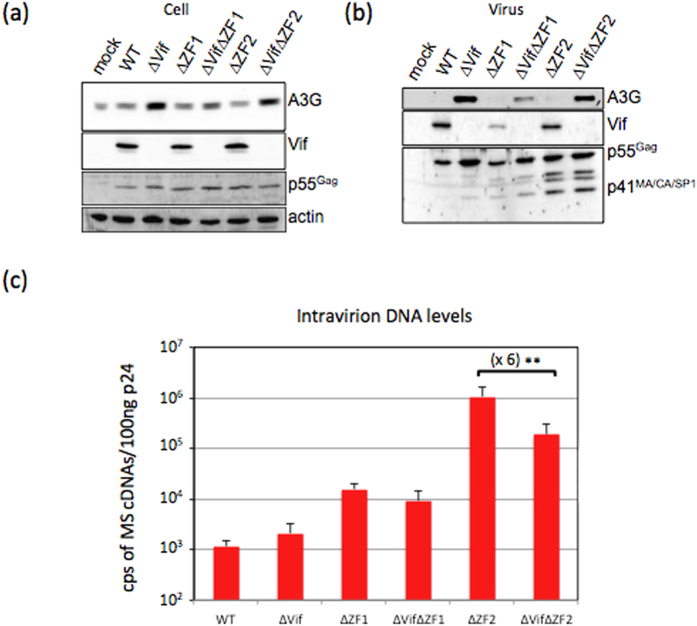
Contribution of cellular A3G restriction factor in late RT. (**a**) Vif-induced A3G degradation in the established HEK 293T cell line expressing A3G. Representative WB experiment showing Gag, Vif and endogenous A3G levels in cells producing WT or mutant HIV-1 constructs. The input cell lysates were monitored with an anti-actin antibody. (**b**) Virion analysis. Gag, Vif and A3G proteins were detected by immunoblotting, as described in Material and Methods. (**c**) Levels of intravirion DNA. Virions released from A3G stable cells line were quantitated by p24 ELISA and viral DNA content of WT and mutant virions (100 ng p24) were measured by qPCR. Data are representative of at least three independent experiments. ^**^P < 0.01.

Vif function in RT is cell-type dependent. When viruses were produced by a permissive cell line (i.e. HEK 293T) and infected either permissive or restrictive cells (as example T-lymphocyte, macrophage, H9 T cells…), the RT of WT and ∆Vif HIV-1 proceeded with apparent similar efficiencies. However, when ∆Vif viruses were produced by restrictive cells, the RT of ∆Vif was strongly impaired irrespective of the target cells identity^21^. Indeed, the permissivity of the producer cell relies on the presence or not of a restriction factor, A3G, counteracted by Vif. In the absence of Vif, A3G of restrictive cells induces viral DNA deamination during RT followed by DNA degradation or impaired viral replication (reviewed in^21^,^50^). Beside its deaminase-activity, A3G reduces the NC-mediated annealing of tRNA to the PBS^51^. When present, Vif directly binds to A3G and counteracts its antiviral activities by inducing its degradation^52^, impairing A3G translation, and thus preventing A3G packaging. Since A3G-Vif relationship plays a crucial role during RT, it was important to examine the effect of Vif in the presence of A3G. Because the late RT activity might vary with cell types, we considered to keep the same cell-type (HEK 293T) and to derive a stable cell line expressing A3G. Then, the HEK 293T:A3G established cell line was transiently transfected with WT or mutant HIV-1 vectors. Protein analysis of cell lysates (Fig. 4a) and released virions (Fig. 4b) by WB showed decreased levels of A3G when Vif was present, irrespective of the NC construct, indicating that deletions in NC did not impair Vif-induced A3G degradation.

In addition to its role in RT, A3G could also interfere with HIV-1 assembly process. Therefore, we searched for DNA presence in virus population. Released viruses were monitored by quantitative p24 ELISA and their viral DNA content was determined by qPCR as described above (Fig. 4c). Results were similar to those observed in absence of A3G (Fig. 2b), indicating that A3G did not influence the function of Vif and NC in late RT.

### Deletions in Psi resulted in the activation of late RT despite the presence of functional NC

**Figure 5:**
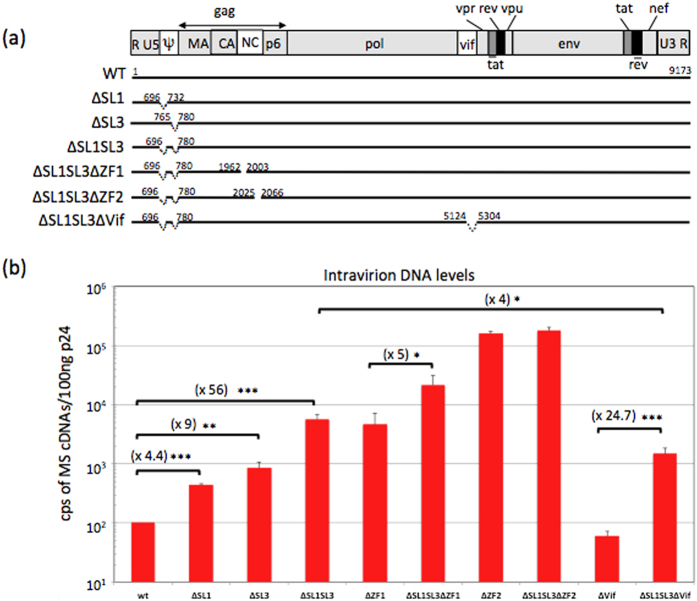
Role of SL1 and SL3 motifs of gRNA in late RT. (**a**) Schematic representation of HIV-1 mutants. Positions of the deletion were numbered according to NL4-3 isolate. (**b**) Levels of intravirion DNA. Viruses produced by 293T cells transfected with WT and mutant HIV-1 constructs were quantitated by p24 ELISA. Viral MS DNA content in 100 ng p24 virions was analyzed by qPCR as in Fig. 4. ^*^^*^^*^P < 0.001, ^*^^*^P < 0.01; ^*^P < 0.05.

To get further insight into the mechanism of the late RT, we asked whether the gRNA must retain its ability to be recognized by Gag NC and packaged into viral particles to be copied into cDNA. The gRNA was deleted from structural motifs in its 5′UTR important for its packaging, such as SL1 and SL3 (Fig. 5a). These motifs belong to the Psi and are recognized by the NC domain of Gag with high affinity^32^,^53^,^54^,^55^. After transfection of HEK 293T cells with these WT or mutant constructions, released virions were analyzed as described above. First of all, the deletion of SL1 or SL3 induced an increase of intravirion DNA levels compared to WT HIV-1, reaching a maximal increase (56-fold) when SL1 and SL3 were deleted together (Fig. 5b). In this latter (∆SL1SL3), intravirion DNA reached the same level as the one obtained by deleting the proximal ZF of NC (∆ZF1). An even higher DNA level could be obtained by combining the ZF1 and Psi deletions (∆SL1SL3∆ZF1), but without reaching the high DNA level of ∆ZF2 mutant. Interestingly, the SL1SL3 deletion has no effect on the DNA content of the ∆ZF2 mutant, probably because RT was already completed and/or reached a plateau when ZF2 is absent (Fig. 5b). Interestingly, the data obtained when normalizing the DNA levels to the genomic RNA levels (Supplemental Fig. S1b), lead to similar interpretation, indicating the absence of bias due to gRNA packaging abilities. Altogether, these data showed that late RT could be activated by mutating either the ZFs of NC or the Psi sequences, indicating that the integrity of SL1SL3 motif as well as the ZFs are required to restrict the late RT. Restriction by SL1SL3 was also observed in the absence of Vif, since the deletion of SL1SL3 in the ∆Vif context induced a 24-fold increase of the DNA content of ∆SL1SL3∆Vif particles compared to ∆Vif (Fig. 5b), suggesting that SL1SL3 impacted on NC rather than on Vif activities. In line with these observations, when we examined the influence of Vif in the absence of SL1SL3, we found that the DNA amount measured in the ∆SL1SL3∆Vif particles still remained 4-times lower than that in ∆SL1SL3 virions. These results supported the fact that Vif did not require SL1SL3 to activate the late RT. In contrast, NC requires the integrity of its primary RNA binding site to restrict RT, as shown by the deletions of ZF motifs or Psi.

## Discussion

HIV-1 reverse transcription is a complex mechanism that has to be spatiotemporally regulated throughout virus replication in cells. Early after virus entry, the RT is efficient while later during late steps of virus assembly, RT has to be restricted to avoid that DNA synthesis starts before virus release thus producing DNA-containing virus. Of course, the RT activity is sensitive to its environment and the composition of the RT complex (RTC) could vary when taking place during early or late steps of replication. A well-known example consists in the antiviral defense mechanism of restrictive cells in which the antiviral A3G factor targets the RT after virus entry and was counteracted by Vif. Here, we have focused on the viral RNA genome and two of its chaperoning proteins, NC and Vif. These three viral components are packaged into infectious viral particles. Thus, they are present together during early virus disassembly and late assembly processes and thereby can potentially interfere into the spatiotemporal control of RT.

Our results revealed for the first time a dynamic connection between NC, the gRNA *via* its Psi region and in a lesser extent Vif, in modulating the late RT activity. In accordance to previous studies, we observed stronger stimulation of the late RT by deleting the NC ZF2 (∆ZF2) than deleting ZF1 (∆ZF1). Similar discrepancies between the two ZFs were also observed with subtle substitutions in ZFs^23^,^24^, confirming that the two ZFs contributed differently to RT regulation. ZF2 is important for the restriction of RT, since its presence prevented the achievement of maximal RT activity (e.g. ∆ZF1). The ZF1 motif plays a pivotal role. ZF1 could reinforce (45-fold) the inhibitory function of ZF2 (∆ZF1 versus WT) to confer optimal inhibitory activity to WT NC. But when ZF2 was missing and thereby the late RT restriction abolished, NC ZF1 in the presence of Vif promoted the RT which achieved optimal levels (as observed with ∆ZF2 and ∆SL1SL3∆ZF2 mutants).

Altogether our results with the deletions in Psi in the context of WT or deleted NC support the notion that NC functions require a functionally competent gRNA, and each ZF contributes differently to gRNA interactions. Indeed, deleting SL1 and SL3, the major RNA binding sites of NC^56^, increased by 56-fold the WT level of DNAs into virions. Also, the ZF2-mediated restriction decreased by 5-fold when SL1 and SL3 were deleted (∆ZF1 versus ∆SL1SL3∆ZF1), suggesting that ZF2 binds to SL1SL3. However, it cannot be excluded that deleting SL1 and/or SL3 hairpins had a more global impact on the structure of the gRNA. Precisely, Webb *et al*. showed that binding of Gag∆ZF2 to Psi RNA was impaired with a Kd decreased by 300-fold compared to that of WT Gag whereas ∆ZF1 Gag binds to Psi RNA with similar affinity as WT Gag *in vitro*^53^. Also, a recent NMR study of the nucleic acid binding properties of NC proposed a task specialization of each ZF in which ZF2 binds first to nucleic acid^57^.

Unlike Psi and NC, Vif function is subtler and was mainly revealed in the context of defective ∆ZF2 NC, as the absence of Vif decreased by 7-fold the ∆ZF2 level of RT. The presence of Vif and Gag ZF1 (∆ZF2) together were always associated with maximal RT suggesting that Vif might interact with NC ZF1 to promote the late RT. These results are consistent with our *in vitro* RT assays showing that addition of Vif to NC ∆ZF2 increased the yield of elongated product. Like NC, Vif binds the 5′ UTR of gRNA^10^,^38^,^39^,^58^ targeting to TAR and polyA stem-loop structures as primary strong binding sites and downstream structures including SL1 and SL3 with moderate affinity^38^. However, Vif-RNA binding can be displaced by the addition of Gag *in vitro*^58^. Thus, the two Gag and Vif proteins, which colocalize in cells^58^,^59^, could compete for their interaction with gRNA.

Interestingly, the function of Vif in late RT is similar in the presence or the absence of A3G, indicating it is independent of its role in counteracting A3G restriction. This implication of Vif in the activation of the late RT might explain why mutating GagNC of a simple gammaretrovirus, MuLV, did not cause the accumulation of a high level of viral DNA in MuLV particles, since MuLV does not express Vif chaperon protein^60^.

Besides the key implications of our data regarding the different interactions within the RTC during late step of virus replication, a better understanding of the spatiotemporal control of the RT would be required to understand the cause and the significance of the late RT. For instance, it might provide new insights into the mechanism leading to the prevalence of DNA-containing HIV-1 in the semen compared to blood of seropositive patients^61^,^62^,^63^.

## Methods

### Plasmids

HIV-1 plasmids used in this study were based on the molecular clone pNL4-3. The ∆ZF1 and ∆ZF2 variants have already been described in^25^ (Fig. 1a), ∆SL1, ∆SL3 and ∆SL1SL3 were described in^34^ (Fig. 5a) and the ∆Vif plasmid in^64^ (Fig. 1a). The double NC-Vif mutants were constructed by swapping a SpeI-AgeI fragment containing the NC sequence between pNL4-3∆Vif and pNL4-3∆ZF1 or pNL4-3∆ZF2 vectors to give pNL4-3∆Vif ∆ZF1 and pNL4-3∆Vif ∆ZF2, respectively. The same restriction fragments were also introduced into the pNL4-3∆SL1SL3 vector to generate the double Psi-NC mutants: pNL4-3∆SL1SL3∆ZF1 and pNL4-3∆SL1SL3∆ZF2. Similar strategy was used to construct the double Psi-Vif mutant (pNL4-3∆SL1SL3∆Vif) by substituting the AatII-SpeI fragment of pNL4-3∆SL1SL3 into pNL4-3∆Vif.

### Cell culture and transfections

HEK 293T cells were grown in DMEM medium (Dulbecco’s modified Eagle’s medium) supplemented with glutamine (2 mM), penicillin (100 U/ml), streptomycin (100 mg/ml) and heat-inactivated fetal calf serum (10% v/v) at 37 °C. To establish the stable cell-line expressing A3G, 293T cells were co-transfected with the pCMVhA3G^65^ and pcDNA3.1-Hygro plasmids at a 1 : 0.1 ratio. Hygromycin selection was started 48 h after transfection and maintained until several hundred of hygromycin resistant clones were selected, pooled and spread in new dishes.

In standard transfection experiments, 3 × 10^6^ cells were grown in T75 flask. The next day, 8 μg of DNA plasmid (supplemented with empty plasmid when necessary) were transfected by phosphate calcium precipitation. In all cases, 6 h after the transfection, cells were trypsinized, extensively washed with fresh medium, centrifuged and transferred in a new flask in order to eliminate the untransfected DNA plasmid at the cell surface and in the medium. The virion-containing supernatant was harvested 48 h after transfection, centrifuged at 1,500 rpm during 10 min at 4 °C, filtered (0.45 μm) and stored at −80 °C. Cells were collected by pipetting with PBS, washed three times with cold PBS 1X and centrifuged 5 min at 1,500 rpm at 4 °C before storage at −80 °C.

### Virus, proteins and DNA extraction

Virions were purified from cell-free culture media by ultracentrifugation through a 20% sucrose cushion at 30,000 rpm in a SW32 rotor for 1 h 30 min at 4 °C. Pellets were resuspended in 160 μl of DMEM with 8 U of RNase-free DNase (RQ1, Promega). Two μl of virion samples were used for virion quantification by using a HIV-1 CAp24 core antigen enzyme-linked immunosorbent assay (ELISA) Kit (Innogenetics), according to the manufacturer’s protocol and a 25 μl aliquot for western-blot analysis. The rest of virions was incubated at 37 °C for 45 min to reduce contamination by the transfecting DNA plasmid. Then, 44 μl of TES 4X (200 mM Tris-HCl pH 7.5, 20 mM EDTA, 0.4% SDS) and 20 μg of tRNA carrier were added to virions before extraction of nucleic acids by phenol/chloroform and ethanol precipitation. Cellular DNA was extracted with DNAzol (MRC) according to the manufacturer’s instructions. Nucleic acids were quantitated by measuring optical absorption at 260 nm. For protein analysis, cells were lysed in the presence of protease inhibitor cocktail (Roche) with the ProteoJet reagent according to the manufacturer’s instructions (Fermentas). Total protein concentration was determined by Bradford assay using a BSA standard set (Fermentas). Equal amount of viruses (equivalent to 1/12 concentrated culture fluid) and cells were loaded on 12% SDS/PAGE and were transferred on nitrocellulose membrane. HIV-1 Gag was detected with either a mouse anti-CA antibody (1/150 of hybridoma H183) or a rabbit anti-MA (1/200, AIDS Reagent Program #4811). Horseradish peroxidase-conjugated (HRP) anti-mouse IgG (diluted at 1/2000, Santa Cruz) or anti-rabbit (1/2000, Santa Cruz) was used as secondary antibody, respectively. Vif was detected with a Vif monoclonal antibody (1/500, AIDS Reagent Program #319) and a secondary antibody HRP-anti-mouse (1/2000). Endogenous A3G was detected with anti-A3G (1/10,000, AIDS Reagent Program (ApoC17) and a secondary anti-rabbit-HRP (1/3000, Santa Cruz). Cellular actin was detected with an anti-actin (1/500, Sigma) and a secondary anti-rabbit HRP-antibody (1/2000). ECL fluorescence was recorded by a CCD chemiluminescence camera system (Gnome, Syngene).

### Quantitation of DNA and RNA

One aliquot of nucleic acids extracted from virions was saved to quantitate gRNA. To this end, RNA were incubated with RQ1 DNase (Promega) in presence of RNaseOUT (Invitrogen) during 25 min at 37 °C and extracted with phenol-chloroform then chloroform and finally precipitated with ethanol 100% and washed with ethanol 70%. RNA pellets were dissolved in water and quantitated by measuring optical absorption. Intravirion RNA were reverse transcribed using an oligo(dT) primer with the Expand RT (Roche). A control experiment was systematically performed without RT to control the absence of DNA contamination as previously described^66^. To monitor viral RNA and MS cDNA, quantitative PCR assay was achieved with 125 ng of tRNA-equivalent virions or 125 ng of cellular DNA samples extracted from cells transfected with either wt or mutant pNL4-3 plasmids, or with empty plasmid as controls (mock). The qPCR was achieved with the SYBR Green kit (Roche) using a RotorGene (Labgene) system. A standard curve was generated from 10^2^ to 10^6^ copies of pNL4-3 plasmid. Nucleic acid level in virions and producer cells was normalized with respect to CAp24 protein (determined by ELISA) and GAPDH gene, respectively^25^,^26^. Sequences of primers and detailed PCR conditions were the same as previously used^25^,^26^ and will be provided on request.

### Recombinant Vif and synthetic NC proteins

Recombinant Vif protein was purified as already described^65^,^67^. Briefly, expression plasmids pD10WTVif was used to transform Escherichia coli BL21 cells, and production of Vif proteins was induced by addition of 0.5 mM IPTG to log phase bacterial cultures (OD_600 nm_ = 0.4–0.6). After 6 h at 22 °C, bacteria were harvested by centrifugation at 4000 × g during 15 min, lysed in the denaturing lysis buffer (6 M guanidine hydrochloride, 100 mM sodium phosphate, 10 mM Tris-HCl, pH 8) at room temperature and stirred overnight. Cellular debris was separated by centrifugation at 27,000 × g during 30 min at 4 °C and the cleared lysate was loaded onto a Ni-NTA agarose column (Invitrogen). The column was washed with the lysis buffer and elution was performed by decreasing pH values (from pH 6.5 to pH 4.5). Fractions containing Vif proteins were analyzed on 12% SDS-PAGE and pooled. Proteins were then renatured by slow dialysis (during 24 h) against buffers with decreasing guadinium chloride concentration, and finally against a buffer containing 50 mM MOPS pH 6.5, 150 mM NaCl, 10% glycerol. Protein was stored at −80 °C before use.

For NC proteins (NL4-3 isolate), the syntheses were performed by solid phase peptide synthesis on a 433A synthesizer (ABI, Foster City, CA), using the standard fluorenylmethoxycarbonyl method with a 0.1 mmol scale^68^,^69^. Amino acids were purchased from PolyPeptide (Strasbourg, France) and Glutamine Wang LL resin from Activotec (Cambridge, UK). The elongation was carried out by coupling of a 10-fold excess of Fmoc-L-amino acid derivatives, using 2-(1*H*-benzotriazol-1-yl)-1,1,3,3-tetramethyluronium hexafluorophosphate (HBTU), 1-hydroxybenzotriazole (HOBt), and diisopropylethylamine (Hünig’s base) (DIEA) as coupling reagents in *N,N*-dimethylformide (DMF) as solvent. After each coupling step, Fmoc deprotection was performed by treatment with 20% piperidine in DMF followed by UV at 301 nm. Cleavage and deprotection of the peptidylresin were performed for 2 h using a 10 ml trifluoroacetic acid (TFA) solution containing water (5%, v/v), TIS (iPr3SiH, 2.5%, v/v) and *1,2-Ethanedithiol* (EDT, 2.5%, v/v). Peptides were purified by HPLC on a C18 column (StrategyN100, 5 μm; 250 × 10, Interchim, France) in water/acetonitrile mixture containing 0.05% TFA with linear gradient from 0 to 45% of acetonitrile for 60 min and monitored at 220 nm. The peptide purity and molecular weight were checked by LC/MS (m/z_cal_/m/z_obt_MMobt NCp7 = 5135/5137; m/z_cal_/m/z_obt_ NCp7∆ZF1 = 4956/4957; m/z_cal_/m/z_obt_ NCp7∆ZF2 = 4770.5/4772.46).

### *In vitro* RT assays

For *in vitro* RT assays, reactions were performed as previously described^12^. Briefly, the first step was to obtain the primer/template complex. Annealing reactions were performed using 1 pmole of RNA 1-278 (HIV-1 pNL4-3 isolate) and 0.3 pmole of 5′-end [^32^P] labeled ODN (1 million cpm) complementary to the PBS region. Primer and template were denaturated 2 min at 90 °C and cooled on ice for 2 min. After addition of 0.1 M NaCl, primer/template samples were incubated 20 min at 50 °C, and kept on ice. In a second step, NC and/or Vif proteins (at indicated concentrations) were incubated with 380 nM HIV-1 RT for 4 min at 37 °C in buffer E2 (0.1 M NaCl, 50 mM Tris-HCl pH 8.3, 6 mM MgCl_2_, 50 μM dNTPs, 1 mM DTT). Reverse transcription was then initiated by addition of pre-incubated primer/template complexes and proceeded for 5 to 40 min at 37 °C. Polymerization was blocked by addition of 10 mM EDTA, 1% SDS and 2 mg/ml proteinase K. After proteolysis for 1 h at 37 °C, samples were phenol/chloroform extracted and precipitated with ethanol. Nucleic acids were resuspended in urea-containing loading buffer and analyzed by denaturing 8% PAGE.

## Additional Information

**How to cite this article**: Racine, P.-J. *et al*. Requirements for nucleocapsid-mediated regulation of reverse transcription during the late steps of HIV-1 assembly. *Sci. Rep*. **6**, 27536; doi: 10.1038/srep27536 (2016).

## Supplementary Material

Supplementary Information
