# Supplementary material for: Requirements for nucleocapsid-mediated regulation of reverse transcription during the late steps of HIV-1 assembly
Source: Sci Rep. 2016 Jun 7;6:27536. doi: 10.1038/srep27536 (PMC4895152; doi:10.1038/srep27536)
Supplement: Supplementary Information [file srep27536-s1.pdf]

Supplementary data

Requirements for nucleocapsid-mediated regulation of reverse transcription during the late steps of HIV-1 assembly

Pierre-Jean Racine[1+], Célia Chamontin[1], Hugues de Rocquigny[2], Serena Bernacchi[3], Jean-Christophe Paillart[3] and Marylène Mougel[1,*]

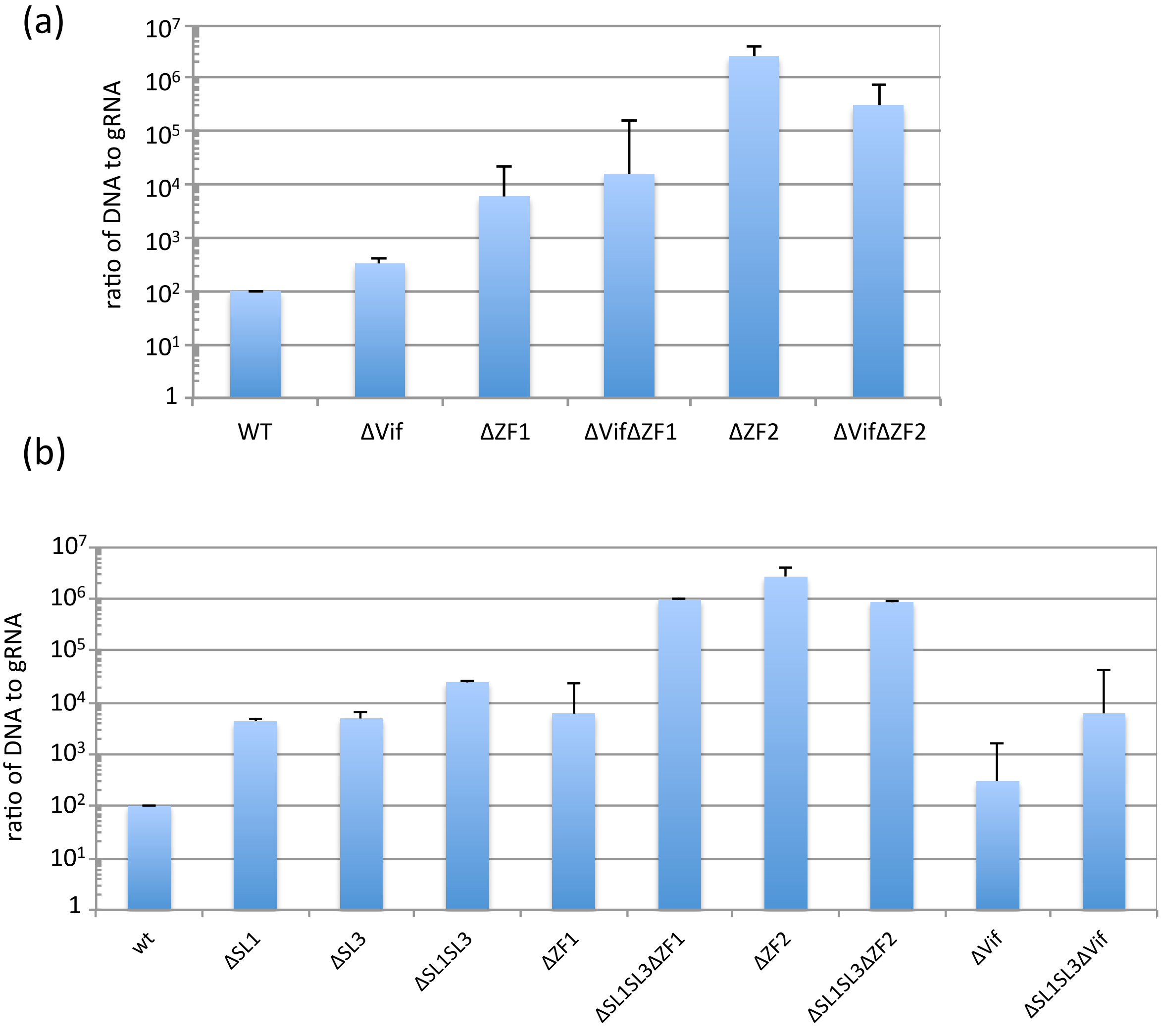

Figure S1: Quantitation of intravirion HIV-1 nucleic acids. Relative amounts of intravirion MS cDNAs and gRNA for the Vif (A) and Psi (B) mutant viruses. Intravirion levels of gRNA and MS DNAs were determined by RT-qPCR and qPCR, respectively (n=2). Number of copies of MS cDNAs and gRNA were determined in the same virus samples, normalized to WT amounts and ratios calculated as (% MS cDNA) / (% gRNA) x 100.
